# Reduced Expression of Hippocampal GluN2A-NMDAR Increases Seizure Susceptibility and Causes Deficits in Contextual Memory

**DOI:** 10.3389/fnins.2021.644100

**Published:** 2021-04-09

**Authors:** Maria Florencia Acutain, Jordana Griebler Luft, Cecila Alejandra Vazquez, Bruno Popik, Magalí C. Cercato, Alberto Epstein, Anna Salvetti, Diana A. Jerusalinsky, Lucas de Oliveira Alvares, Maria Verónica Baez

**Affiliations:** ^1^Instituto de Biología Celular y Neurociencia “Prof. E. De Robertis” (IBCN, CONICET-UBA), Buenos Aires, Argentina; ^2^Instituto de Biociências, Universidade Federal do Rio Grande do Sul, Porto Alegre, Brazil; ^3^CGMC, University Lyon I, Lyon, France; ^4^International Center for Infectiology Research (CIRI), INSERM U1111, CNRS UMR5308, Université de Lyon (UCBL1), Lyon, France; ^5^1° U.A. Departamento de Histologia, Embriología, Biologia Celular y Genética, Facultad de Medicina, Universidad de Buenos Aires, Buenos Aires, Argentina

**Keywords:** NMDA receptors, GluN2A subunit, GluN2A/GluN2B, mRNA silencing, long-term memory, seizure induction, hippocampus

## Abstract

*N*-methyl-D-aspartate receptors are heterotetramers composed of two GluN1 obligatory subunits and two regulatory subunits. In cognitive-related brain structures, GluN2A and GluN2B are the most abundant regulatory subunits, and their expression is subjected to tight regulation. During development, GluN2B expression is characteristic of immature synapses, whereas GluN2A is present in mature ones. This change in expression induces a shift in GluN2A/GluN2B ratio known as developmental switch. Moreover, modifications in this relationship have been associated with learning and memory, as well as different pathologies. In this work, we used a specific shRNA to induce a reduction in GluN2A expression after the developmental switch, both *in vitro* in primary cultured hippocampal neurons and *in vivo* in adult male Wistar rats. After *in vitro* characterization, we performed a cognitive profile and evaluated seizure susceptibility *in vivo*. Our *in vitro* results showed that the decrease in the expression of GluN2A changes GluN2A/GluN2B ratio without altering the expression of other regulatory subunits. Moreover, rats expressing the anti-GluN2A shRNA *in vivo* displayed an impaired contextual fear-conditioning memory. In addition, these animals showed increased seizure susceptibility, in terms of both time and intensity, which led us to conclude that deregulation in GluN2A expression at the hippocampus is associated with seizure susceptibility and learning–memory mechanisms.

## Introduction

*N*-methyl-D-aspartate receptors (NMDAR) are one of the most important ionotropic receptors responsible for glutamatergic excitatory transmission in the brain, mainly because they are considered the postsynaptic molecular coincidence detector of presynaptic and postsynaptic activity (Wigström and Gustafsson, 1986; Kandel et al., 2012). NMDAR are heterotetramers composed by two GluN1 obligatory subunits that are necessary for receptor assembly at the endoplasmic reticulum and two regulatory subunits codified by four grin2 genes (GluN2A, GluN2B, GluN2C, or GluN2D) and two grin3 genes (GluN3A and GluN3B). Each regulatory subunit confers different physiological and pharmacological properties to the assembled functional NMDAR (Paoletti et al., 2013). It is well established that GluN2A and GluN2B are the main NMDAR regulatory subunits expressed in the hippocampus and cortical areas (Lau and Zukin, 2007; Yashiro and Philpot, 2008; Paoletti et al., 2013; Sanz-Clemente et al., 2013; Shipton et al., 2014), and their expression is tightly regulated in a temporal pattern during development and synapse maturation.

GluN2B is highly expressed throughout embryonic life. After birth, GluN2B transcription and translation start to decrease and are expressed at lower levels during adult life. On the other hand, GluN2A has the lowest basal levels during embryonic period, and its expression rises in the course of early postnatal development, reaching a plateau during adult life (Sans et al., 2000; Paoletti et al., 2013). The change in GluN2A-GluN2B expression during early postnatal development is called developmental switch (Sans et al., 2000; Lau and Zukin, 2007; Yashiro and Philpot, 2008; Sanz-Clemente et al., 2013; Shipton et al., 2014), which is also observed *in vitro*, in cultured neurons (Corbel et al., 2015). As a result of this switch, an increase in the GluN2A/GluN2B ratio is observed in mature neurons; this relationship is commonly used as a parameter of synapse and circuit maturation. Furthermore, different alterations in the GluN2A/GluN2B ratio are observed during several learning and memory paradigms and also after exposing an animal to stimulus enrichment (Kopp et al., 2007; Jacobs et al., 2015; Holehonnur et al., 2016; Bessières et al., 2019). Recently, several studies have attempted to elucidate the role of each subunit and the relationship between GluN2A and GluN2B subunits in physiological processes and neuropathological conditions (Bannerman, 2009; Yuan et al., 2014; Kumar, 2015).

Mutations in grin2A and grin2B (the genes that encode for GluN2A and GluN2B, respectively) are associated with a discrete range of neuropathologies. Grin2B mutations are associated with developmental disorders and autism spectrum disorders (ASDs). On the other hand, familiar and *de novo* grin2A mutations that induce either gain or loss of NMDAR function have been described in patients with complex neurological disorders including the development of seizures and in some cases cognitive impairment and ASD (Endele et al., 2010; Lemke et al., 2013; Pierson et al., 2014; Yuan et al., 2014; Burnashev and Szepetowski, 2015; Swanger et al., 2016; Addis et al., 2017; Sibarov et al., 2017; Cardis et al., 2018; Punnakkal and Dominic, 2018; Strehlow et al., 2019; Amador et al., 2020; Mota Vieira et al., 2020). In order to investigate a possible association between grin2A mutations and patient phenotype, several studies have attempted to establish whether mutation sites are located within domains involved in subunit structure and receptor channel function (Addis et al., 2017; Sibarov et al., 2017; Strehlow et al., 2019). In addition, it has been shown that some grin2A mutations caused a reduction in GluN2A expression and also in synaptic plasticity (Addis et al., 2017; Amador et al., 2020; Mota Vieira et al., 2020).

The effects of GluN2A knockdown have been studied to a limited extent only (Sepulveda et al., 2010; Gambrill and Barria, 2011; de Solis et al., 2015). Sepulveda et al. showed that reduced GluN2A expression did not alter GluN2B levels, which resulted in a decreased GluN2A/GluN2B ratio (Sepulveda et al., 2010). Furthermore, Sepulveda et al. (2010) and Gambrill and Barria (2011) described that neurons where GluN2A was expressed at lower levels showed increased dendritic spine density compared to controls (Sepulveda et al., 2010; Gambrill and Barria, 2011). Nonetheless, in both cases, GluN2A silencing was induced before the developmental switch, blocking the rise in GluN2A expression and the consequent developmental maturation processes. More recently, it was shown that a reduction in GluN2A expression induced *in vivo*, at the amygdala and after the developmental switch, caused memory impairment in animals exposed to a fear-conditioning paradigm (de Solis et al., 2015). Nevertheless, in the cases where GluN2A was knocked out (KO), no changes in several long-term memory tasks were observed (Bannerman et al., 2008, 2012; Bannerman, 2009).

Taking into account these antecedents, we hypothesized that the reduction in GluN2A expression after the developmental switch could modify the GluN2A/GluN2B ratio and consequently lead to a change in behavior and seizure susceptibility. In this study, we reduced GluN2A expression levels using a specific shRNA anti-GluN2A [shGluN2A, codified by an adeno-associated vector (AAVe)] after the developmental switch both *in vitro*, in primary cultured hippocampal neurons, and *in vivo*, in young adult male Wistar rats. After *in vitro* characterization, we performed a cognitive profile and evaluated seizure susceptibility in hippocampal injected rats. Our results showed that the reduction in GluN2A expression did not induce an increase in GluN2B levels as a compensatory mechanism, resulting in a decrease in the GluN2A/GluN2B ratio. In the cognitive profile, shGluN2A-expressing rats showed unaltered object recognition memory. However, these animals presented changes in spatial exploration and an impairment in contextual fear conditioning. Additionally, these animals also showed an increase in seizure susceptibility, both in time and intensity, suggesting that the decrease in GluN2A expression in a mature system would be associated with seizure onset and contextual learning–memory acquisition.

## Materials and Methods

### Viral Vector Stocks

The AAVe encoding a specific shRNA against GluN2A (AAVe-shGluN2A, shGluN2A: 5′-GAACGTGGATGTCGGATCCTT 3′) (Gambrill and Barria, 2011) or a scrambled sequence without a known target in mammalian cells (AAVe-shsc, shsc: 5′-ACGTGACACGTTCGGAGAATT-3′) was as previously described (Ploquin et al., 2013). Each shRNA sequence (Gambrill and Barria, 2011) was cloned into the *Bbs*I site between the ITR sequences in the pBS-GFP-AAV-U6 plasmid. Viral stocks were produced by cotransfection with three plasmids: the pBS-GFP-U6-shGluN2A or pBS-GFP-U6-shsc, the helper, and the rep-cap plasmids in HEK 293SZ cells. The cells and supernatant were collected 48 h posttransfection, and viral stocks were purified by CsCl gradient and quantified by flow cytometry (Ploquin et al., 2013).

### Neuronal Cultures

Hippocampal neuronal cultures were performed from Wistar embryos on day 17 (E17) (Kaech and Banker, 2007), with some modifications (Cercato et al., 2017). Briefly, both hippocampi were dissected from E17 pups and digested with trypsin (Sigma, Sigma–Aldrich Co., St. Louis, MO, United States). Cells were plated onto poly-L-lysine (Sigma)–coated glass coverslips (Waldemar Knittel Glasbearbeitungs GmbH, Germany) or into coated wells and incubated in neurobasal media (NB; Invitrogen, Life Technologies Corporation, Carlsbad, CA, United States) supplemented with B27 (Invitrogen) and Glutamax (Invitrogen). Cultures were maintained in complete NB (NB + B27 + Glutamax) at 37°C and with 5% CO_2_. Culture media were replaced by 1/3 every 2 days.

At 13 days *in vitro* (DIV), neuronal cultures were transduced with the AAVe-shGluN2A or the AAVe-shsc vectors diluted in conditioned complete NB with a multiplicity of infection (MOI) of 1 × 10^4^. After transduction, neuronal cultures were maintained in complete NB at 37°C and with 5% CO_2_ for 1 to 8 days postinfection (DPI).

### Immunofluorescence

For immunofluorescence (IF) assays, coverslips were first fixed with 4% paraformaldehyde–4% sucrose solution for 10 min. Then, cultures were permeabilized in 0.1% Triton X-100–phosphate-buffered saline (PBS) and blocked with 5% normal goat serum in 0.05% Tween-20 PBS (TPBS). After blocking, coverslips were incubated with the primary antibodies for 1 h at 37°C, washed with TPBS, and then incubated with Cy3 and/or Alexa Fluor 647–conjugated secondary antibodies (1:300) for 30 min at 37°C. After washing with TPBS, coverslips were mounted using Mowiol (MOWIOL 4-88 Reagent, Sigma–Aldrich).

The following antibodies were used: anti-GluN1 (mouse monoclonal, 1:300, BD Pharmingen), anti-GluN2A (rabbit polyclonal, 1:100, Millipore), anti-GluN2B (mouse polyclonal, 1:100, BD Pharmingen), anti-rabbit–Alexa Fluor 647 (goat polyclonal, 1:300, Abcam), anti-mouse–Cy3 (goat polyclonal, 1:300, Thermo Fisher Scientific).

### Image Analysis

Images from IF assays were obtained under a spinning disc Olympus-IX83 microscope (Olympus Co., Tokyo, Japan). Each image was taken under the 60 × objective, with a 1.42 numerical aperture oil objective and an ORCA-Flash4.0 V2 Digital CMOS camera, using CellSens software (Olympus) with customized filter sets. A Z stack of 0.2 μm step was performed in each channel using the same exposure time. Images were analyzed with Fiji software (ImageJ, NIH, Bethesda, MD, United States). For every assay, a max projection was performed, and mean fluorescence was determined in 10 to 15 neurons/culture from three separate and independent cultures. To evaluate changes in NMDAR subunits, all the images were subjected to an absolute defined intensity threshold for each protein mark and related to the fluorescence obtained in the shsc-transduced culture for the same protein. All analyses were performed blind to treatment conditions.

### Animals and Injections

A total of 40 male Wistar rats (2–3 months old, weighing between 300 and 400 g) from CREAL, Federal University of Rio Grande do Sul (UFRGS), were used in behavioral and seizure susceptibility assays. Rats were housed in Plexiglas boxes (four animals per cage), with block randomization using the cage as a subgroup to ensure that each cage contained at least one animal per experimental group. Animals were kept on a 12-h light–dark cycle under controlled temperature (22°C ± 2°C), with regular chow and water available *ad libitum* and humidity of approximately 65%. All experiments were performed during the light cycle (Popik et al., 2020). The animals were handled for 3–5 days before they were assayed. All procedures followed the Brazilian ethical guidelines for animal research, set by the National Council for the Control of Experimental Animal Research (CONCEA), and in accordance with the guidelines of the US National Institutes of Health Guide for the Care and Use of Laboratory Animals (NIH publication no. 8023, revised 1978). Methods and results are reported according to the revised ARRIVE guidelines (Percie, du Sert et al., 2020).

Vector infusions were performed in Wistar male rats under intraperitoneal (i.p.) anesthesia with ketamine (75 mg/kg) and xylazine (10 mg/kg). Meloxicam was administered (1 mg/kg; via subcutaneous injection) as an analgesic and anti-inflammatory 20 min before surgery, as well as once a day on the following 2 days. The AAVe were injected bilaterally (MOI: 2 × 10^4^, 2 μL/side) at a rate of 0.07 μL/min in the CA1 region of the dorsal hippocampus (from bregma: AP -4.0 mm, LL ± 3.0 mm, DV -1.8 mm) (Paxinos and Watson, 2007). Half of the animals were infused with the AAVe-shGluN2A, and the other 20 rats, of similar weight, with the AAVe-shsc control vector. After surgery, both groups of animals were taken back to their home cages with food and water *ad libitum*. They were allowed 7 days to recover and be handled before any further experiments were carried out.

We evaluated GluN2A expression levels in a pilot experiment, to ensure reduction of GluN2A levels during behavioral assays. In each animal, the injection site was identified by direct scar visualization after animal decapitation, and transgene vector expression was confirmed by enhanced green fluorescent protein (eGFP) mRNA levels in the injection area.

### Behavioral Assays

**FIGURE 1:**
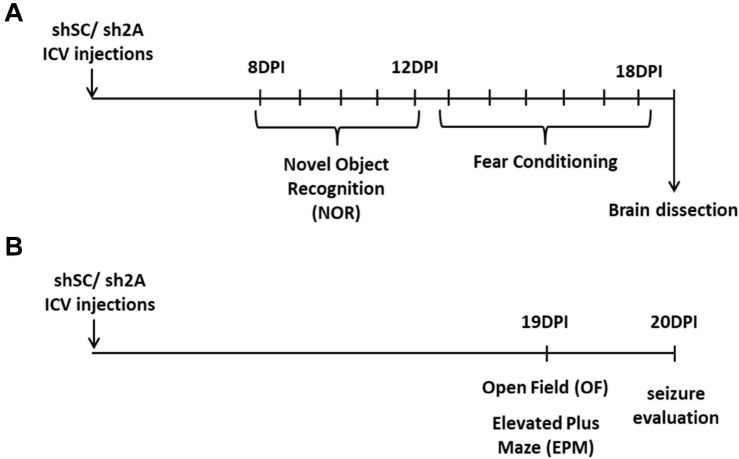
Experimental design. **(A)** One group of animals was injected bilaterally with AAVe-shsc (control) or AAVe-shGluN2A (shGluN2A) in the hippocampal CA1 region. After 8 DPI, rats were evaluated in novel object recognition (NOR) up to 12 DPI. Then, animals were assayed in contextual fear-conditioning tasks. At 19 DPI, animals were euthanized, and the hippocampi collected to perform mRNA extraction. **(B)** In a second group of animals, injected either with shsc (control) or shGluN2A AAVe (shGluN2A), anxiety-like behavior was evaluated. At 19 DPI, these rats were exposed to an open field (OF) and then to an elevated plus maze (EPM). At 20 DPI, seizure-induced susceptibility was assessed in these animals by administration of PTZ or saline, as a vehicle.

Assays were conducted with two groups of animals. One group of animals injected either with the AAVe-shsc (*n* = 10) or the AAVe-shGluN2A (*n* = 10) vector was exposed to the following behavioral tasks: at 8 DPI, rats were exposed to a novel object recognition (NOR) task up to 12 DPI. Then, from 13 to 18 DPI, shGluN2A- and shsc-expressing animals were trained and tested in a fear-conditioning task. After the behavioral tasks, at 19 DPI, animals were euthanized by decapitation, the injection location was visualized, and the hippocampi extracted to analyze NMDAR subunit levels (Figure 1).

Another group of 20 animals was injected either with the AAVe-shsc (*n* = 10) or the AAVe-shGluN2A (*n* = 10). At 19 DPI, injected rats were exposed to an open field (OF) and 4 h later, to an elevated plus maze (EPM, Figure 1) to evaluate locomotion, stress, and anxiety-like behavior. The following day (20 DPI), these animals were tested in a pharmacologic seizure induction assay.

Each behavioral assay was performed and recorded by an experimenter who was blind to the AAVe injections and then analyzed offline. Then, a second analysis was performed by another experienced operator that received only the list of videos. In case of differences between both analyses, a third evaluation was carried out by a different investigator.

### Open Field

Locomotor activity was evaluated in the OF test at 19 DPI. AAVe-shsc– or AAVe-shGluN2A–injected rats were exposed to the arena for 5 min. The OF consisted of a 50-cm-high, 60 × 40-cm plywood box, with two striped black and white walls (one vertical and one horizontal) and two black walls with different white visual clues. Exploring behavior was recorded by video tracking and processed offline with Tox Trac Software (Rodriguez et al., 2018). During the analysis, distance traveled, average speed, and exploration rate were evaluated by the software. In addition, a central square area covering 60% of the arena was defined to determine the center and periphery areas of the arena. Then, the time that animals spent in each area was measured. Rearing behavior was also assessed. After the OF session, animals were returned to their home cages until the next task.

### Elevated Plus Maze

The EPM consisted of four 40 × 10-cm arms, two open and two closed, elevated 50 cm above the ground. The day of the experiment, animals were left in the testing room for 30 min, to induce habituation, before the assay. Then, shGluN2A- and shsc-transduced rats were allowed to explore the EPM for 5 min. Each experiment was recorded with a video camera for further analysis. Exploration time and entries, either into the open or closed arms, were quantified by an experienced observer. Afterward, rats were returned to their home cages until the next day.

### Novel Object Recognition

Rats were exposed to an OF for 10 min per day on 3 consecutive days, to induce habituation (experimental scheme on Figure 4A). On the fourth day, two similar objects (A-A’) were added to the OF. Then, rats were left to freely explore them for 5 min (training session), and the time that the animal spent exploring each object was recorded. After training, rats were returned to their home cages. On the fifth day, these rats were exposed to the familiar and a novel object for 5 min (A, B; test session). Discrimination index (DI) was calculated as follows: time exploring novel object - time exploring familiar object/time exploring novel object + time exploring familiar object (Cercato et al., 2017).

The objects used for this task were similar in texture and size (i.e., about 10 cm high and 7 cm wide) but had distinctive shapes (i.e., cubes and hemispheres). Objects and positions were counterbalanced across experiments and behavioral trials.

### Fear Conditioning

The auditory fear-conditioning test was performed as previously described (Popik et al., 2020), with some modifications. The task consisted of a 6-consecutive-day assay in two different contexts (A and B). The conditioning chambers used were illuminated Plexiglas boxes with the same dimensions (33 × 25 × 25-cm grid of parallel 0.1-cm-caliber stainless steel bars spaced 1 cm apart), but with different walls. Context A had black walls, whereas context B had vertically black and white striped walls.

On the first 2 days, shGluN2A- and shsc-transduced rats were habituated to conditioning chamber A (10 min each day, Figure 5A). On the third day, rats were placed in context A for 2 min before receiving the conditioning stimulus: 30-s presentation of a 5-kHz, 75-dB tone (CS) that was paired with 1-s 0.5-mA foot shock. The paired CS was presented three times during this session, with an interpairing interval of 1 min (conditioning session). Then, rats were kept in the conditioning context for an additional 2 min before returning to their home cages with water and food *ad libitum*. On the fifth day, animals were deposited in conditioning chamber B and exposed to three CS, without foot shock (auditory test). Finally, 24 h later, on the sixth day, rats were re-exposed to chamber A for 2 min (contextual test).

Freezing behavior was measured as a memory index (time freezing) and registered using a stopwatch in real time by an experienced observer that was unaware of the experimental group. Freezing was defined as total immobility except that required for breathing (Blanchard and Blanchard, 1969) and was scored in three blocks of 30 s both in the test and renewal session. Moreover, contextual freezing was measured for the first 2 min before exposure to the CS in both sessions.

### Seizure Induction

At 20 DPI, animals were injected i.p. with pentylenetetrazol (PTZ, 50 mg/kg; obtained through a pilot test) (Sigma–Aldrich, St. Louis, MO, United States) or vehicle (saline). Each animal received a single injection, and the induced seizure was recorded for 30 min. The intensity of seizures following PTZ injection was analyzed according to Racine adapted scale (Lüttjohann et al., 2009): stage 1, sudden behavioral arrest; stage 2, facial jerking with mouth and face clonus; stage 3, neck jerks; stage 4, bilateral forelimb clonus in a sitting position; stage 5, forelimb clonus with rearing and falling; and stage 6, generalized seizure while lying on the side with wild jumping. Clonic seizure was considered at stages 3 and 4, and the generalized tonic and/or tonic–clonic seizure was observed at stages 5 and 6.

### mRNA Quantification

After the indicated DPI, total-RNA extraction either from neuronal cultures or hippocampal homogenates from injected animals was performed using ARN-PrepZOL reagent (Inbio-Highway, Argentina) according to the manufacturer’s instructions. Total RNA was resuspended in RNAse-free water, and the concentration and purity of RNA were estimated by measuring the A260 and A280 in a Nanodrop 2000/2000c (Thermo Scientific, United States). Then, 1 μg of total RNA from each region was reverse-transcribed using 200 pmol of hexa-random primers (Genbiotech, United States), 100 nmol of a deoxynucleotide triphosphate (dNTP) mixture, and Moloney murine leukemia virus reverse transcriptase (200 units; Genbiotech, United States). Twenty units of the ribonuclease inhibitor RNAsin (Genbiotech, United States) was added to each reaction tube at a final volume of 30 μL of 1X reverse transcriptase buffer. Reverse transcription reaction was performed at 37°C for 90 min and then maintained at 42°C for 15 min. Reactions were stopped by heating at 80°C for 5 min and cooling on ice. Each reverse-transcribed product was diluted with RNAse-free water to a final volume of 60 μL and stored at −20°C for further use.

cDNA amplification was performed using 5 μL of the obtained cDNA, 10 pmol of each primer (Invitrogen Argentina), and the SYBR^®^ Select Master Mix (Applied Biosystems Inc., Life Technologies, United States) to a final volume of 20 μL. Table 1 shows the primers used to analyze the selected mRNAs, using *cypA* as housekeeping gene. The cDNA levels were determined using a real-time polymerase chain reaction (PCR) system StepOne Cycler (Applied Biosystems Inc., Life Technologies, CA, United States). Product purity was confirmed by dissociation curves, and random samples were subjected to 2% agarose gel electrophoresis. Negative DNA template controls were included in all the assays and yielded no consistent amplification. The threshold cycles (Ct) and PCR efficiency were calculated with Step One software (Applied Biosystems Inc., Life Technologies, United States). For each target, the fold expression over control values was calculated using the relative standard curve methods designed to analyze data from real-time PCR (Čikoš et al., 2007). The relative target quantity for all experimental samples was determined from the standard curve, normalized to the relative quantity of the reference gene, and finally divided by the normalized target value of the control sample. No significant differences in Ct values were observed for *cypA* between the different experimental assays.

**TABLE 1:** Sequences and bp of the selected mRNAs.

**Gene name**	**Accession number**	**Primer sequence**	**Size (bp)**
*cypA*	NM_017101.1	TATCTGCACTGCCAAGACTGAGTG	127
		CTTCTTGCTGGTCTTGCCATTCC	
*grin1*	NM_001270602.1	AACGGGAGTCCAAGGCAGAG	148
		TACACTGTGGCAGCGTCGTC	
*grin2a*	NM_012573.3	CCCAGGCTTGTGGTGATCGT	175
		CGAAGGGGGCTTCCTCCAAG	
*grin2b*	NM_012574.1	TATCTGCACTGCCAAGACTGAGTG	127
		CTTCTTGCTGGTCTTGCCATTCC	
*egfp*	NC_025025.1	TCGTGACCACCCTGACCTA	140
		GTAGTTGCCGTCGTCCTTG	

### Western Blot

After the indicated DPI, AAVe-transfected neuronal cultures were homogenized separately in a Teflon glass potter (5 × 15″), in 100 mM NaCl, 0.2% Triton X-100, 1 mM EGTA, and 20 mM HEPES buffer (pH 7.4) with 1X antiproteases cocktail (Sigma). Protein concentration was measured in a Nanodrop 2000/2000c (Thermo Scientific, United States). Samples were then diluted to a concentration of 2 μg/μL in PBS, resuspended in Laemmli buffer 2X in a 1:1 ratio, and heated for 5 min at 100°C. All samples were individually processed and analyzed. Protein samples were separated on 8% sodium dodecyl sulfate–polyacrylamide gel electrophoresis gel and transferred to a polyvinylidene difluoride membrane (Immobilon-P, Millipore). Blots were blocked with 5% non-fat milk–0.1% Tween-20 in Tris-buffered saline and incubated with primary antibodies: anti-GluN1 (rabbit polyclonal 1:1,000, Sigma), anti-GluN2A (rabbit polyclonal, 1:1,000 Millipore), anti-GluN2B (mouse polyclonal, 1:500 BD Pharmingen), and anti-GAPDH (rabbit polyclonal, 1:5,000, Sigma). After washout, blots were incubated with horseradish peroxidase (HRP)–conjugated anti-rabbit secondary antibody (1:10,000; Amersham Biosciences, GE Healthcare, Piscataway, NJ, United States) or HRP-conjugated anti-mouse secondary antibody (1:10,000; Sigma), developed in SuperSignal West Pico chemiluminescent substrate solution (Thermo Scientific, Waltham, MA, United States), and exposed to film (Agfa-Gevaert NV, Mortsel, Belgium).

To determine the actual amount of each protein, the respective band intensity was measured using ImageJ software (https://imagej.nih.gov) and relativized to the corresponding GAPDH band used as internal control, for each assay.

### Statistical Analysis

For each experiment, variables were first analyzed for Gaussian distribution by Kolmogorov–Smirnov normality test. Then, an analysis was performed either by Student *t* test (checking homoscedasticity by *F* test to compare variances) or by one- or two-way analysis of variance (ANOVA) (using Tukey test to compare variances). One-way ANOVA was followed by posttest analysis via Tukey or Dunnett tests, when appropriate. All experimental data are expressed as mean ± SEM.

For variables that did not adjust to a Gaussian distribution, data were analyzed using non-parametric statistics (Kruskal–Wallis test or Mann–Whitney) and expressed as medians with interquartile ranges. For each set of experiments, the statistical test used is shown in each figure legend. Data analysis was performed using GraphPad Prism 7.0 (GraphPad Software, Inc., San Diego, CA, United States).

## Results

### shGluN2A Transduction Induces a Specific Silencing of GluN2A NMDAR Subunit in Neuronal Cultures

Previous work described that GluN2A KO or silencing prior to the developmental switch induced small changes in neuronal morphology and functionality (Sepulveda et al., 2010; Gambrill and Barria, 2011; Gray et al., 2011; Kannangara et al., 2014). However, in these models, decreased GluN2A expression could be compensated by mechanisms that contribute to the normal development of neurons. To get deep insight on how the downregulation of GluN2A expression affects a mature system, we employed an AAVe carrying a specific shRNA antiGluN2A (AAVe-shGluN2A) or a scrambled sequence as control (AAVe-shsc) (12–14 DIV, Supplementary Figure 1) and generated an *in vitro* model of GluN2A-reduced expression. Neuron survival assays showed no significant differences between cultures infected with either AAVe-shsc (shsc-transduced cultures), AAVe-shGluN2A (shGluN2A-transduced cultures), or non-infected (NI), up to 8 DPI (Supplementary Figure 1).

**FIGURE 2:**
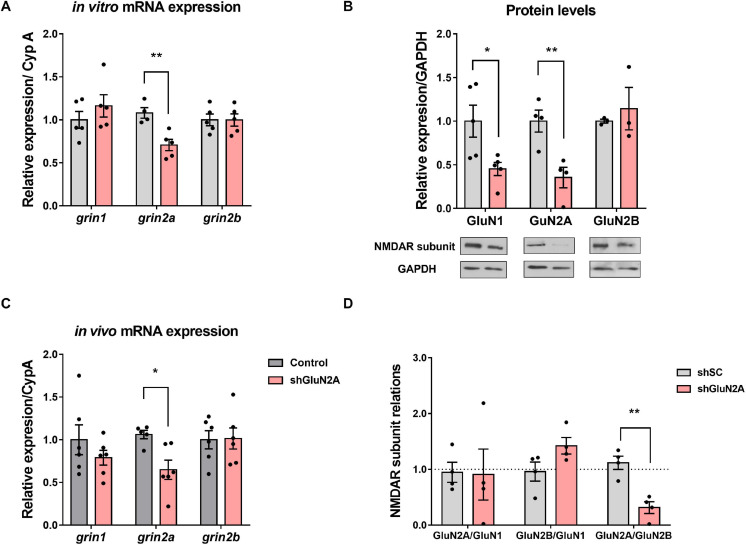
NMDAR expression in the GluN2A-reduced expression *in vitro* model. **(A)** Primary hippocampal neuronal cultures infected with AAVe-shGluN2A showed a significant reduction in *grin2a* mRNA expression (**p < 0.01, unpaired *t* test). However, no significant differences were detected in *grin1* (*p* = 0.3467, unpaired *t* test) or in *grin2b* mRNA (*p* = 0.9922, unpaired *t* test, *n* = 5 independent cultures). **(B)** NMDAR main subunit protein levels at 6 DPI. GluN2A and GluN1 levels were significantly decreased in shGluN2A-transduced cultures compared with the shsc-transduced ones (**p < 0.01, *p < 0.05, unpaired *t* test). GluN2B levels remained similar to controls (*p* = 0.5875, unpaired *t* test, *n* = 5 independent cultures). **(C)** mRNA analysis of NMDAR subunit expression at the hippocampus of AAVe-injected rats at 20DPI. *grin2a* expression was significantly decreased in the hippocampi from GluN2A KD rats (*p < 0.05, unpaired *t* test, *n* = 6), whereas *grin1* and *grin2b* levels (*p* = 0.3057 and *p* = 0.9285, respectively, unpaired *t* test) were similar to control (shsc injected) animals. **(D)** Relative abundance of NMDAR subunits in the primary hippocampal neuronal cultures. There was an imbalance in the ratio between the regulatory subunits GluN2A and GluN2B in the shGluN2A-transduced cultures (**p < 0.01, unpaired *t* test, *n* = 4 independent cultures). On the other hand, GluN2A/GluN1 and GluN2B/GluN1 ratios remained similar to shsc-transduced cultures (*p* = 0.9360 and *p* = 0.0853, respectively, unpaired *t* test, *n* = 4 independent cultures). In all cases, bars represent mean ± SEM.

We then evaluated GluN2A-reduced expression in hippocampal neuronal cultures infected with either AAVe-shsc or AAVe-shGluN2A from 1 to 8 DPI. At each DPI, cultures were homogenized, and RNA was isolated and analyzed by reverse transcriptase–quantitative PCR (RT-qPCR). In these assays, *grin2A* expression decreased gradually from 2 to 5 DPI only in shGluN2A-transduced cultures, being significantly different from shsc-transduced cultures at 6 DPI (Supplementary Figure 1B and Figure 2A). No significant changes in *grin1* or *grin2B* expression were detected at any of the DPI analyzed, in shGluN2A- or shsc-transduced cultures (Figure 2A and Supplementary Figures 1C,D).

In parallel, NMDAR subunits were analyzed by Western blot. In this assay, GluN2A protein levels were significantly decreased at 6 DPI only in shGluN2A-transduced cultures (Figure 2B). In these cultures, GluN1 level was also decreased at 6 DPI. On the other hand, no changes in GluN2B levels were detected at least at 6 DPI (Figure 2B). As NMDAR subunit ratio could be considered a hallmark of synapse maturation, we calculated the ratios between GluN1, GluN2A, and GluN2B protein levels. Results showed that, as a consequence of GluN2A reduction, the GluN2A/GluN2B ratio was decreased in shGluN2A-transduced cultures compared with shsc-transduced ones. However, no other subunit ratio was affected (Figure 2D). These results indicated that shGluN2A transduction in hippocampal mature neurons induced a specific decrease of GluN2A expression, without any accompanying change in GluN2B levels. Furthermore, this change in expression altered NMDAR subunit composition and relative abundance, with a decreased GluN2A/GluN2B ratio reflecting a more immature stage.

**FIGURE 3:**
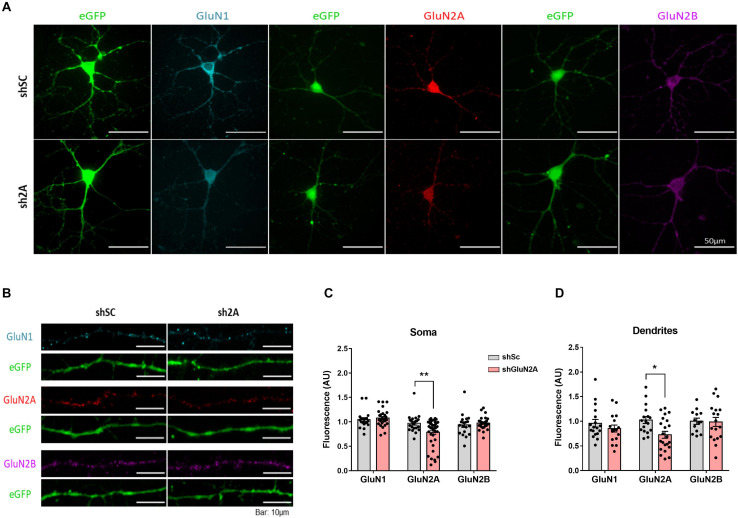
NMDAR cellular distribution in the shGluN2A-transduced cultures. **(A)** NMDAR subunit immunofluorescence of primary hippocampal neuronal cultures transduced either with AAVe-shsc or AAVe-shGluN2A at 6 DPI. **(B)** Representative dendrites for GluN1, GluN2A, and GluN2B immunofluorescence. **(C)** The shGluN2A-transduced cultures showed a significant reduction in GluN2A levels at soma compared with the shsc controls (**p < 0.01, unpaired *t* test). Nevertheless, GluN1 (*p* = 0.5396, unpaired *t* test) and GluN2B (*p* = 0.4897, unpaired *t* test) levels were similar to controls. **(D)** There was a significant decrease in GluN2A level (*p* = 0.0409, unpaired *t* test), without differences in GluN1 (*p* = 0.3145, unpaired *t* test) and GluN2B (*p* = 0.9079, unpaired *t* test), at dendrites. Data are represented as mean ± SEM, *n* = 3 independent cultures.

Considering that a reduction in GluN2A levels could affect NMDAR assembly and distribution, we investigated the subcellular localization of the main NMDAR subunits expressed at the hippocampus. We decreased GluN2A expression in primary hippocampal neuron cultures as described above. In this assay, shsc- and shGluN2A-transduced cultures were fixed and analyzed by IF against GluN1, GluN2A, and GluN2B (Figure 3). As expected, a significant decrease in GluN2A IF levels was observed at soma (Figures 3A,C) and dendrites (Figures 3B,D). We also observed that the reduction of GluN2A levels in this last compartment (0.73 ± 0.07 compared to controls, Figure 3D) was similar to that observed at soma (0.79 ± 0.05 compared to controls, Figure 3C). Unexpectedly, IF analysis showed that GluN1 levels (Figure 3) were similar between shGluN2A- and shsc-transduced cultures in soma (Figures 3A,C), as well as in dendrites (Figures 3B,D). Furthermore, no changes in GluN2B IF levels were observed either at soma or at dendrites (Figures 3C,D).

Altogether, our *in vitro* results showed that reduced expression of GluN2A in hippocampal cultures induces an imbalance in GluN2A/GluN2B ratio, which reflects a more immature phenotype and is accompanied by a decrease of GluN2A-NMDAR both at dendrites and soma.

### GluN2A Reduced Expression *in vivo*

Changes in NMDAR subunit expression *in vivo* have been described during learning and memory processes (reviewed in Baez et al., 2018) and also in neuropathological conditions (Lau and Zukin, 2007; Sanz-Clemente et al., 2013; Burnashev and Szepetowski, 2015). Therefore, to assess the effect of GluN2A-reduced expression *in vivo*, we injected the AAVe-shsc and the AAVe-shGluN2A in the CA1 region of the dorsal hippocampus of adult Wistar rats. In order to test the effectiveness of shGluN2A transduction, expression of NMDAR subunits was measured by RT-qPCR (Figure 2C). In accordance with our *in vitro* results, *grin2A* levels were only decreased in AAVe-shGluN2A–injected animals. Furthermore, no changes in *grin1* or *grin2B* expression were observed in AAVe-shGluN2A– or AAVe-shsc–injected rats (Figure 2D).

#### Decreased Expression of Hippocampal GluN2A Impaired Contextual Fear Memory but Not Object Recognition or Auditory Fear Memory

**FIGURE 4:**
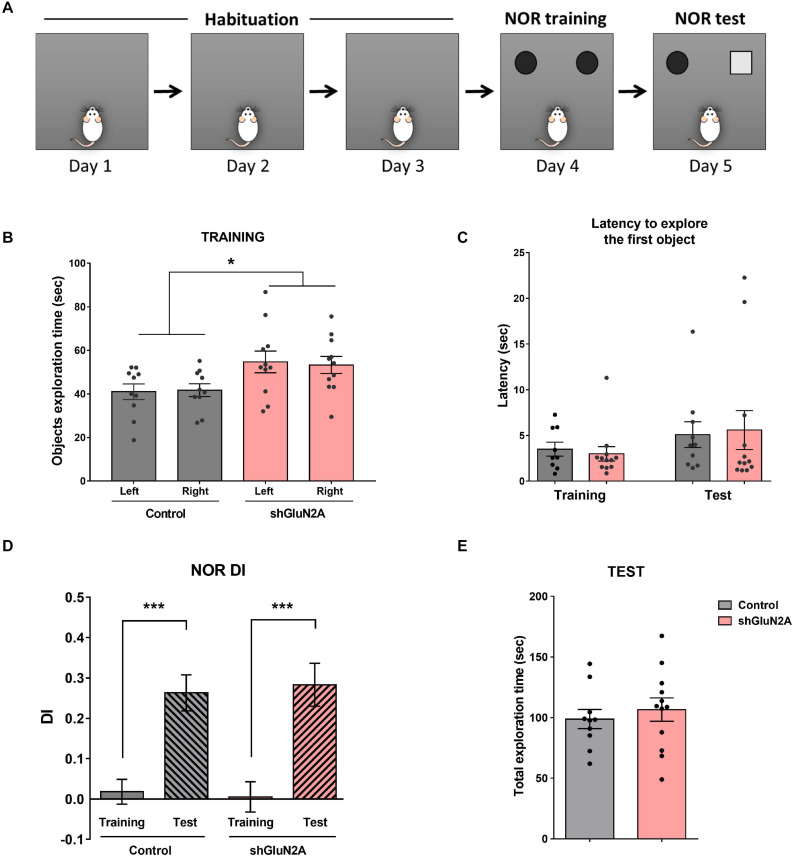
Novel object recognition. **(A)** Representative scheme of the novel object recognition (NOR) task. Habituation to the arena: three 10-min sessions on consecutive days. NOR training session: rats were exposed to two identical objects for 5 min in the familiar arena. NOR test session: rats were exposed to one familiar and one novel object for 5 min in the arena. **(B)** Training session: shGluN2A-injected animals showed a significant increase in total object exploration time (*p < 0.05, Two-way ANOVA, Tukey posttest). However, there was no significant difference between exploration of each object in control and shGLUN2A-injected animals. **(C)** shGluN2A-injected animals showed similar latencies to explore the objects compared to controls during training (*p* = 0.6478, unpaired *t* test) and test sessions (*p* = 0.8527, unpaired *t* test). **(D)** NOR test session: discrimination index (DI) at training (plain bars) and test (striped bars) were represented. Both groups of animals reached learning criteria DI_te_ significantly different from DI_tr_ (***p < 0.0001, unpaired *t* test). **(E)** There was no difference in total object exploration time during the test session between control and shGluN2A-injected animals (*p* = 0.5494, unpaired *t* test). Data are represented as mean ± SEM. Animals: *n* = 9 control, *n* = 11 GluN2A KD.

**FIGURE 5:**
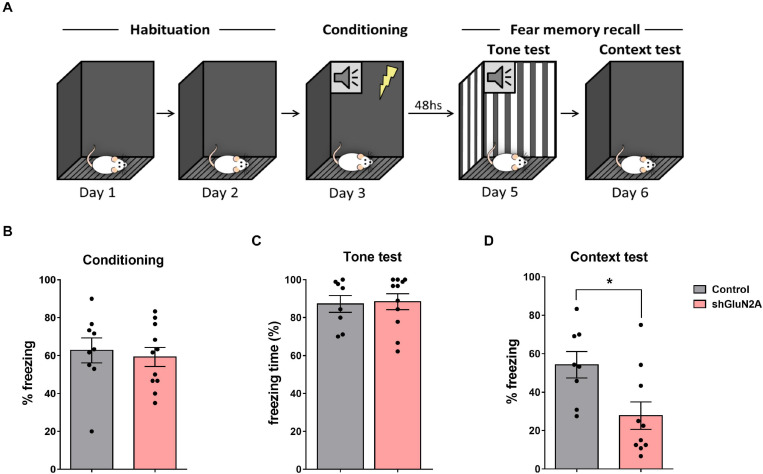
Fear conditioning. **(A)** Representative scheme of the experimental design. Habituation to the context for 10 min on 2 consecutive days. Then, in the conditioning session, the tone (CS) was paired with 0.5-mA foot shock. Tone test: exposure to the tone in a novel context. Context test: exposure to the conditioning context. **(B)** shGluN2A-injected animals showed similar freezing behavior during the conditioning session compared to shsc-injected rats (*p* = 0.6722, unpaired *t* test). **(C)** There was no difference in freezing behavior between control and shGLUN2A-injected animals in the tone test (*p* = 0.8537, unpaired *t* test). **(D)** shGLUN2A-injected rats showed a significant decrease in freezing expression during the context test (*p < 0.05, unpaired *t* test). Data are represented as mean ± SEM. *N* = 9 shsc-injected, *N* = 11 shGLUN2A-injected animals.

In order to evaluate potential memory deficits following reduction of GluN2A expression levels *in vivo*, rats injected with AAVe-shGluN2A or AAVe-shsc at the dorsal hippocampal CA1 region were tested in a series of behavioral tasks (Figures 1, 4, and 5).

First, the animals were trained and tested in a NOR task (Figure 4A). Time exploring objects, latency to exploration of the first object, and DI were analyzed in training and test sessions (Figures 4B–E). During the training session, AAVe-GluN2A–injected rats spent significantly more time exploring both objects, without any significant difference in the time spent exploring each one (Figure 4B). There was no difference in the latency to explore the first object during both training and test sessions (Figure 4C). Furthermore, there was a significant difference between training and test DI in both groups, without significant difference in DI during the test between shGluN2A- and shsc-injected animals (Figure 4D). These results indicated that both groups are able to recognize and discriminate a familiar from a novel object in a similar manner. Also, these results suggested that the reduction of hippocampal GluN2A expression did not induce changes in object recognition memory.

It has been shown that blocking GluN2A-NMDAR with an antagonist disrupted fear memory expression (Gao et al., 2010; Dalton et al., 2012; Zhang et al., 2015). For this reason, we decided to assay a fear memory paradigm that included auditory and contextual components (Figure 5A). In the conditioning session, no significant differences in freezing behavior were observed (Figure 5B). Also, when animals were subjected to the tone test in a different context (context B), no significant differences between shGluN2A-injected and control rats were detected (Figure 5C). Finally, when subjected to the context test in the familiar context A, shGluN2A-expressing rats showed a significant reduction in freezing levels compared to control animals (Figure 5D, context A). These results indicated that reduced GluN2A hippocampal expression induced an impairment/deficit in contextual fear memory.

#### Effects of Reduced Expression of Hippocampal GluN2A on Anxiety and Locomotor Activity

**FIGURE 6:**
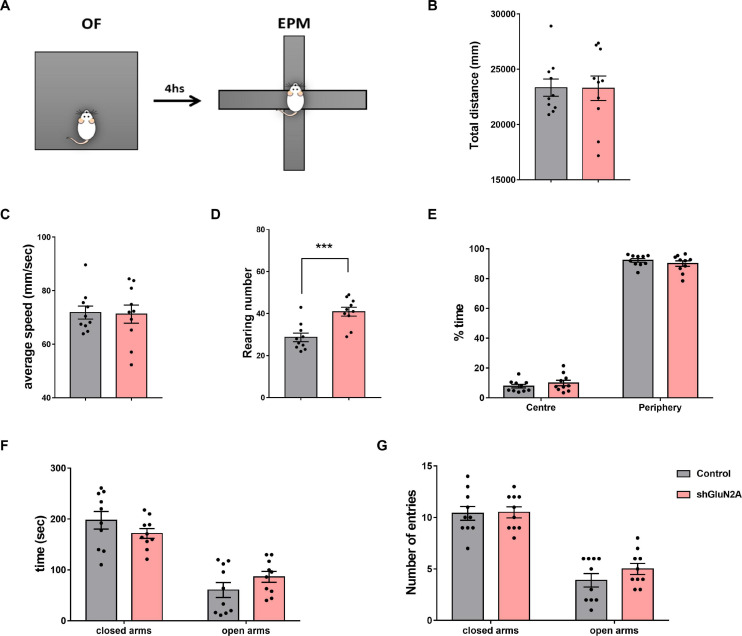
Anxiety-like behavior analysis. **(A)** Experimental design: shGLUN2A- and shsc-injected rats were exposed to an open field (OF) for 5 min, and 4 h later to the elevated plus maze (EPM) task for 5 min **(B–E)** OF analysis. No significant differences were observed in total traveled distance (**B**, Horizontal exploration, *p* = 0.9690, unpaired *t* test) or in locomotor activity (**C**, average speed, *p* = 0.8872, unpaired *t* test) between control and KD animals. **(D)** shGluN2A-injected animals showed a significant increase in vertical exploration (rearing behavior), compared to controls (***p < 0.001, unpaired *t* test). **(E)** There was no difference in region preference (center/periphery) during arena exploration between the two groups (*p* = 0.5711, unpaired *t* test). **(F,G)** EPM analysis. Both groups explored the open and closed arms similarly, measured as time spent in the open or closed arms (**F**, *p* = 0.3161, unpaired *t* test) and number of entries into the arms (**G**, *p* = 0.9089 and *p* = 0.3783, unpaired *t* test). Data are represented as mean ± SEM. Animals: *N* = 10 shsc-injected, *N* = 10 shGLUN2A-injected.

We decided to analyze locomotor activity, stress, and anxiety-like behavior in hippocampal AAVe-shGluN2A–injected rats, as these animals explored both familiar and new objects for a longer time in the NOR training, without impairment in long-term memory expression. Hence, a second group of AAVe-shGluN2A– and AAVe-shsc–injected rats was exposed first to an OF in order to evaluate exploration behavior and stress levels. Then, these rats were assayed in an EPM paradigm to test anxiety-like behavior (Figures 1, 6).

Open field analysis revealed that both shGluN2A- and shsc-injected animals did not show significant difference in horizontal exploration parameters, as distance covered and average speed were similar in both groups (Figures 6B,C). Moreover, no significant differences between GluN2A-reduced expression and control animals were detected in the time spent in the center and periphery of the arena (Figure 6D). However, a significant increase in the number of rearings during the OF task was observed in animals displaying reduced expression of hippocampal GluN2A (Figure 6E).

The EPM paradigm was used to test anxiety-like behavior (Figures 1, 6). In this task, exploration time and entries into the open and closed arms were quantified (Figures 6E–G). No significant differences were observed in the number of entries or in the time spent in open and closed arms between both groups (Figures 6F,G). These results suggest that GluN2A silencing did not alter anxiety-like behavior or locomotor activity levels in shGluN2A-treated animals.

Altogether, results obtained in behavioral assays raise the possibility that reduction of hippocampal GluN2A expression induced an impairment only in tasks involving a contextual component that was visualized as a different form to explore the space in the OF and NOR tasks and also in the reduction in contextual fear long-term memory responses.

#### Reduced Expression of Hippocampal GluN2A Alters Seizure Susceptibility

**FIGURE 7:**
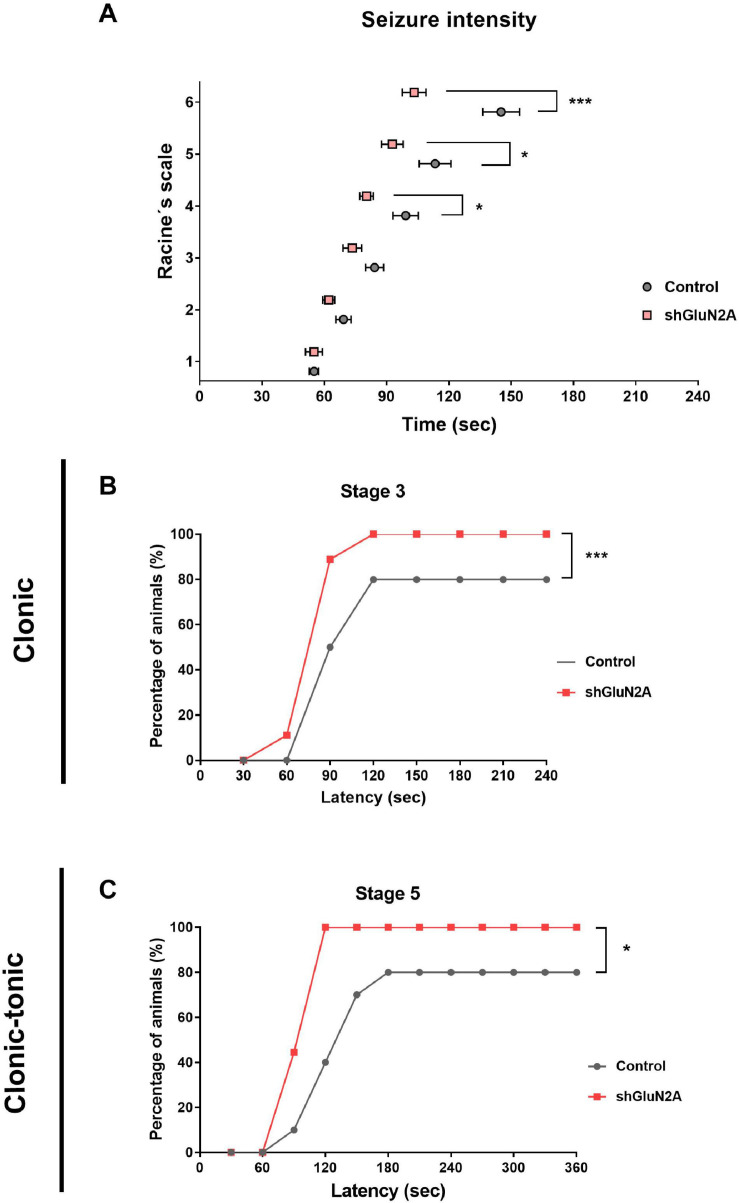
Seizure susceptibility analysis. shGLUN2A- and shsc-injected animals were injected intraperitoneally with a convulsive drug (PTZ, 50 mg/kg) or saline i.p. All animals were monitored after the injection and seizure intensity was evaluated by Racine scale. **(A)** shGLUN2A-PTZ–injected animals reached the highest stages in Racine scale earlier than shsc-PTZ injected ones (*p < 0.05, ***p < 0.001, unpaired *t* test). **(B,C)** There was a significant difference in the percentage of shGLUN2A-injected animals that showed clonic (**B**, ***p < 0.001, two-way ANOVA, Tukey posttest) and clonic–tonic seizure stages (**C**, *p < 0.05, two-way ANOVA, Tukey posttest) compared to the shsc-injected animals. Data are represented as mean ± SEM. PTZ injected animals: *N* = 8 shsc-injected, *N* = 8 shGluN2A-injected.

It has been shown that mutations in *grin2A* induced a decrease in GluN2A expression and led to complex neurodevelopmental disorders that include the occurrence of seizures (Endele et al., 2010; Lemke et al., 2013; Pierson et al., 2014; Yuan et al., 2014; Burnashev and Szepetowski, 2015; Swanger et al., 2016; Addis et al., 2017; Sibarov et al., 2017; Cardis et al., 2018; Punnakkal and Dominic, 2018; Strehlow et al., 2019; Amador et al., 2020; Mota Vieira et al., 2020). In order to evaluate whether the decrease in GluN2A expression is associated with susceptibility to the development of seizures, shGluN2A- and shsc-transduced rats were treated with the convulsive drug PTZ in order to induce acute generalized seizures (Wu et al., 2006; Amada et al., 2013; Sefil et al., 2014; Alachkar et al., 2018). At 20DPI, shGluN2A- and shsc-transduced rats were injected either with saline or PTZ (50 mg/kg i.p.) and monitored for 30 min (Lüttjohann et al., 2009; Sefil et al., 2014; Samokhina and Samokhin, 2018). In each rat, seizure severity was analyzed according to Racine adapted scale (Lüttjohann et al., 2009). In these assays, we observed that shGluN2A-transduced rats injected with PTZ showed a lower seizure threshold than control PTZ-injected rats (Figure 7A). By analyzing tonic and tonic–clonic stages, we found that animals displaying a reduced expression of GluN2A showed higher seizure severity scores and decreased latency time post-PTZ stimulus (Figures 7B,C). Furthermore, we found a significant difference in the number of shGluN2A-injected animals that reached the first tonic and tonic–clonic stage of the seizure (stages 3 and 5, respectively) (Figures 7B,C). As expected, saline-injected rats transduced either with AAVe-shsc or AAVe-shGluN2A did not show any signs of seizure (data not shown). These results suggested that decreased expression of GluN2A facilitated seizure susceptibility and raised the possibility that the decrease in GluN2A expression in a mature system would be directly associated with the development of seizures.

## Discussion

In this work, we silenced GluN2A expression using a specific shRNA both *in vitro*, in cultured hippocampal neurons, and *in vivo*, in a young adult Wistar rat model. As silencing was induced when GluN2A levels were higher than GluN2B in both models (Sans et al., 2000; Lau and Zukin, 2007; Yashiro and Philpot, 2008; Corbel et al., 2015), compensatory mechanisms deployed before the occurrence of the developmental expression switch would be prevented. Moreover, localized reduction of GluN2A expression provided important insights into the role of GluN2A in hippocampal function in a mature system. This would also help to understand the relevance of this subunit in normal rodent behavior, as well as in learning and memory processes.

Our *in vitro* results showed that reduced expression of GluN2A induced a decrease in GluN1 protein levels; although not mRNA levels, this suggests that the decrease in GluN1 is induced at posttranscriptional levels. Regulation of GluN1 expression has been largely evaluated at the transcriptional level (Zukin and Bennett, 1995; Bradley et al., 2006; Yeh et al., 2008; Paoletti et al., 2013; Sanz-Clemente et al., 2013), a few reports showed a posttranscriptional regulation of grin1 mRNA (Anji and Kumari, 2006, 2011; Vedeler et al., 2012) and GluN1 (Abe et al., 2004; Zeng et al., 2019) stability. Our results showed that *grin1* mRNA levels in shGluN2A-reduced expression cultures are similar to those of controls, at least up to 8 DPI (and also *in vivo*, at the analyzed time points). Future experiments evaluating different grin1-RNA binding proteins and their regulation should be carried out to elucidate this possible mechanism.

Other studies have shown that the levels of functional NMDARs present in neurons are determined by the availability of GluN2 subunits (Wenthold et al., 2003; Bradley et al., 2006). Our results confirm that the decrease in GluN2A expression led to a decrease in GluN1 levels, which could be considered a direct measurement of the NMDAR amount (Paoletti et al., 2013). However, we were not able to discriminate whether the decrease in GluN1 and GluN2A levels were localized to a specific subcellular compartment. These suggests that once reduction of GluN2A expression is induced after the developmental switch, neurons would try to maintain the amount of functional and mature NMDAR using the remaining GluN2A and GluN1 subunits in the assembly of new NMDARs.

In all analyzed cases, our results showed that reduced expression of GluN2A did not cause changes in GluN2B expression at mRNA and protein levels *in vitro*. This observation was similar to those models where a knockdown (Sepulveda et al., 2010) or KO of GluN2A (Strehlow et al., 2019) were induced before the developmental switch. Altogether, these results indicate that GluN2A and GluN2B expressions are regulated independently of each other, regardless of when silencing was induced. Moreover, no compensatory expression of GluN2B was induced in the absence of GluN2A transcription/translation. As a result, reduction of GluN2A expression and the lack of change in GluN2B levels led to a decrease in the GluN2A/GluN2B ratio, which was previously associated with more immature phenotypes as the absence of GluN2A did not allow for synapse maturation.

The decrease in GluN2A hippocampal expression did not induce gross behavioral abnormalities, and we did not detect any difference in the stress and anxiety status between shGluN2A- and shsc-injected expressing rats. However, animals showed an enhancement in vertical exploration during the OF task and increased object exploration time during the NOR training, which led us to hypothesize that these animals explore the space or acquire the contextual information in a different way. Amador and colleagues generated a grin2A transgenic mouse expressing a grin2A mutant variant with reduced levels of GluN2A expression and a gain of function: these mice showed a complex neurological phenotype, with the most prominent features being hyperactivity, decreased anxiety-like, and repetitive behaviors (Amador et al., 2020). Altogether, these results raise the possibility that the extent in time and space of changes in GluN2A expression is responsible, at least in part, for behavioral abnormalities.

There is controversy about GluN2A silencing and memory processes. There is evidence that GluN2A KO does not alter spatial long-term memory acquired after several training sessions over days (Bannerman et al., 2008; Bannerman, 2009; Franchini et al., 2020). However, other studies described an impairment in a fear-conditioning task both in a GluN2A knockdown model (de Solis et al., 2015) and after blocking GluN2A-NMDAR in the amygdala (Dalton et al., 2012). Moreover, it has been described that a single training session is sufficient to alter GluN2A expression during memory consolidation (Cercato et al., 2017; Baez et al., 2018; Franchini et al., 2019). In the present study, we analyzed recognition and context-fear learning and memory in two different paradigms in order to evaluate the hippocampal role of GluN2A in each task. In the NOR task, animals were able to recognize the novel object presented in the test. However, shGluN2A-injected animals explored the objects longer during training than the controls. These results differ from those obtained by knocking out IQGAP1, a scaffold protein present in diverse signaling pathways at postsynaptic level. It was described that IQGAP1^–/–^ animals show a decrease in GluN2A levels at synaptosome fractions and that they present impairment in object location and object recognition paradigms, without any difference in exploration during training (Gao et al., 2011). However, as IQGAP1 interacts with multiple effectors, not only with GluN2A, long-term memory deficits could result from the alteration of multiple pathways.

Although it is well established that the hippocampus plays an essential role on contextual fear conditioning, but not in auditory fear conditioning (Phillips and LeDoux, 1992; Sacchetti et al., 1999), its role in object recognition task is still a matter of debate. Several studies have demonstrated that the hippocampus mediates object recognition memory (Cohen et al., 2013; Cercato et al., 2017; Furini et al., 2020), whereas others have found that the hippocampus supports object location, but not object recognition memory (Mumby et al., 2002; Hardt et al., 2010; Warburton et al., 2013; Balderas et al., 2015). It is possible that the participation of the hippocampus in some specific processes but not others might explain why we have found memory impairment in the contextual fear conditioning but not in object recognition and auditory fear-conditioning tasks.

Our results showed that the reduction in hippocampal GluN2A expression induced impairment in the contextual component of the fear-conditioning task, whereas tone-dependent fear conditioning remained unaltered. Also, it is possible that the auditory fear memory was not affected because it was assessed with less of a delay than context-cued memory. On the other hand, it has recently been shown that a set of neurons that are activated by context-fear conditioning is located in the ventral hippocampus and connects monosynaptically to neurons in the basal amygdala (Kim and Cho, 2020). These results appear contradictory because we induced silencing of GluN2A expression in the dorsal but not in the ventral hippocampus. However, previous studies indicate that contextual fear learning strengthens a subset of dorsal hippocampal CA3–CA1 synapses, which is in agreement with the data presented here (Choi et al., 2018). Furthermore, our results are consistent with previous reports that evaluate the role of GluN2A in this kind of task (Kiyama et al., 1998; Gao et al., 2010; Dalton et al., 2012; de Solis et al., 2015) and together strengthen the idea that hippocampal GluN2A is critical for contextual learning (Kiyama et al., 1998; Bannerman et al., 2008).

*Grin2A* mutations that induce both a gain and a loss of function of the NMDAR were associated with complex syndromes that include, to a great extent, the occurrence of seizures (Endele et al., 2010; Lemke et al., 2013; Pierson et al., 2014; Yuan et al., 2014; Burnashev and Szepetowski, 2015; Swanger et al., 2016; Addis et al., 2017; Sibarov et al., 2017; Cardis et al., 2018; Punnakkal and Dominic, 2018; Strehlow et al., 2019; Amador et al., 2020; Mota Vieira et al., 2020). More recently, it has been shown that some *grin2A* mutant variants induced a decrease in GluN2A expression (Addis et al., 2017; Amador et al., 2020; Mota Vieira et al., 2020). In this work, we focused on the relationship between decreasing GluN2A expression and seizure onset. For this reason, we chose PTZ injection, as it is a well-established model of acute generalized seizure (Barker-Haliski and Steve White, 2020). We found that reduced expression of GluN2A at the hippocampus increased seizure susceptibility in the assayed model. Animals injected with shGluN2A showed higher seizure severity scores and decreased latency time post-PTZ stimulus. Furthermore, all shGluN2A-injected animals showed the tonic–clonic stage of seizures, which was not achieved by all the control rats.

These results led us to hypothesize that reduced GluN2A expression followed by the decrease in GluN2A/GluN2B ratio would block synapse maturation as this process requires the change in NMDAR subunit composition from GluN2B-NMDAR to GluN2A-NMDAR and triheteromeric NMDAR containing both regulatory subunits [reviewed in Lau and Zukin (2007); Sanz-Clemente et al. (2013), and Shipton et al. (2014)]. Reducing GluN2A expression prevents this change and also leads to a slight decrease in GluN1 translation and reduced expression of NMDAR. These decreases have been reported to destabilize spine structure and over time lead to loss of spines and excitatory synapses (Alvarez et al., 2007), which in turn would cause neurons to establish new connections with limited maturation (mainly due to reduced expression of GluN2A). The increased proportion of neurons in a more immature state, characterized by a rearrangement in gene expression that ultimately results in altered cell morphology (Sepulveda et al., 2010; Gambrill and Barria, 2011; Kannangara et al., 2014) and changes in spines (Kannangara et al., 2014), is comparable to that observed in processes that, *in vivo*, originate seizures. Further experiments should be carried out to elucidate the role of GluN2A in the mature brain and shed light onto the mechanisms underlying seizure susceptibility caused by downregulation of GluN2A expression.

Notwithstanding the attention that GluN2A has received in the field during the last few years, this is the first work that assessed seizure susceptibility not associated with a particular mutation found in patients. Several reports have evaluated the effects of different *grin2A* mutant variants on NMDAR function, revealing a possible relationship between altered expression and/or trafficking of GluN2A and the mutation responsible for the phenotype observed (Addis et al., 2017; Strehlow et al., 2019; Amador et al., 2020; Mota Vieira et al., 2020). Hence, the present study shows a potential link between seizure susceptibility and reduced expression of hippocampal GluN2A in a mature system and the relevance of this downregulation in spatial learning and memory processes.

## Data Availability Statement

The raw data supporting the conclusions of this article will be made available by the authors, without undue reservation.

## Ethics Statement

The animal study was reviewed and approved by National Council for the Control of Experimental Animal Research (CONCEA), Brazil.

## Author Contributions

MA did and analyzed the experiments and also collaborated with manuscript writing. JG, CV, BP, and MC did the experiments. AS and AE assesorated with the AAV vectors. DJ was the director of the DEVENIR laboratory where the vectors were designed and built. LO was the director of the behavioral work and collaborated with manuscript writting. MB designed and directed the work, did and analyzed the experiments, and wrote the manuscript. All authors contributed to the article and approved the submitted version.

## Conflict of Interest

The authors declare that the research was conducted in the absence of any commercial or financial relationships that could be construed as a potential conflict of interest.

## References

[B1] Abe M. Fukaya E. M. Yagi T. Mishina M. Watanabe M. Sakimura K. (2004). NMDA receptor GluRε/NR2 subunits are essential for postsynaptic localization and protein stability of GluRζ1/NR1 subunit. *J. Neurosci.* 24 7292–7304. 10.1523/jneurosci.1261-04.2004 15317856 PMC6729774

[B2] Addis L. Virdee J. K. Vidler L. R. Collier D. A. Pal D. K. Ursu D. (2017). Epilepsy-associated GRIN2A mutations reduce NMDA receptor trafficking and agonist potency-molecular profiling and functional rescue. *Sci. Rep.* 7:66. 10.1038/s41598-017-00115-w PMC5427847 28242877

[B3] Alachkar A. Łażewska D. Latacz G. Frank A. Siwek A. Lubelska A. (2018). Studies on anticonvulsant effects of novel histamine h3r antagonists in electrically and chemically induced seizures in rats. *Int. J. Mol. Sci.* 19:3386. 10.3390/ijms19113386 30380674 PMC6274786

[B4] Alvarez V. A. Ridenour D. A. Sabatini B. L. (2007). Distinct structural and ionotropic roles of NMDA receptors in controlling spine and synapse stability. *J. Neurosci.* 27 7365–7376. 10.1523/jneurosci.0956-07.2007 17626197 PMC6672602

[B5] Amada N. Yamasaki Y. Williams C. M. Whalley B. J. (2013). Cannabidivarin (CBDV) suppresses pentylenetetrazole (PTZ)-induced increases in epilepsy-related gene expression. *PeerJ* 1:e214. 10.7717/peerj.214 24282673 PMC3840466

[B6] Amador A. Bostick C. D. Olson H. Peters J. Camp C. R. Krizay D. (2020). Modelling and treating GRIN2A developmental and epileptic encephalopathy in mice. *Brain* 143 2039–2057. 10.1093/brain/awaa147 32577763 PMC7363493

[B7] Anji A. Kumari M. (2006). A novel RNA binding protein that interacts with NMDA R1 mRNA: regulation by ethanol. *Eur. J. Neurosci.* 23 2339–2350. 10.1111/j.1460-9568.2006.04776.x 16706842

[B8] Anji A. Kumari M. (2011). A cis-acting region in the N-methyl-d-aspartate R1 3′-untranslated region interacts with the novel RNA-binding proteins beta subunit of alpha glucosidase II and annexin A2 - effect of chronic ethanol exposure in vivo. *Eur. J. Neurosci.* 34 1200–1211. 10.1111/j.1460-9568.2011.07857.x 21995826 PMC3195980

[B9] Baez M. V. Cercato M. C. Jerusalinsky D. A. (2018). NMDA receptor subunits change after synaptic plasticity induction and learning and memory acquisition. *Neural Plast.* 2018:5093048. 10.1155/2018/5093048 PMC5863338 29706992

[B10] Balderas I. Rodriguez-Ortiz C. J. Bermudez-Rattoni F. (2015). Consolidation and reconsolidation of object recognition memory. *Behav. Brain Res.* 285 213–222. 10.1016/j.bbr.2014.08.049 25192636

[B11] Bannerman D. M. (2009). Fractionating spatial memory with glutamate receptor subunit-knockout mice: figure 1. *Biochem. Soc. Trans.* 37 1323–1327. 10.1042/bst0371323 19909269

[B12] Bannerman D. M. Bus T. Taylor A. Sanderson D. J. Schwarz I. Jensen V. (2012). Dissecting spatial knowledge from spatial choice by hippocampal NMDA receptor deletion. *Nat. Neurosci.* 15 1153–1159. 10.1038/nn.3166 22797694 PMC3442238

[B13] Bannerman D. M. Niewoehner B. Lyon L. Romberg C. Schmitt W. B. Taylor A. (2008). NMDA receptor subunit NR2A is required for rapidly acquired spatial working memory but not incremental spatial reference memory. *J. Neurosci.* 28 3623–3630. 10.1523/jneurosci.3639-07.2008 18385321 PMC6671075

[B14] Barker-Haliski M. Steve White H. (2020). Validated animal models for antiseizure drug (ASD) discovery: advantages and potential pitfalls in ASD screening. *Neuropharmacology* 167:107750. 10.1016/j.neuropharm.2019.107750 31469995 PMC7470169

[B15] Bessières B. Jia M. Travaglia A. Alberini C. M. (2019). Developmental changes in plasticity, synaptic, glia, and connectivity protein levels in rat basolateral amygdala. *Learn. Mem.* 26 436–448. 10.1101/lm.049866.119 31615855 PMC6796789

[B16] Blanchard R. J. Blanchard D. C. (1969). Passive and active reactions to fear-eliciting stimuli. *J. Comp. Physiol. Psychol.* 68 129–135. 10.1037/h0027676 5793861

[B17] Bradley J. Carter S. R. Rao V. R. Wang J. Finkbeiner S. (2006). Splice variants of the NR1 subunit differentially induce NMDA receptor-dependent gene expression. *J. Neurosci.* 26 1065–1076. 10.1523/jneurosci.3347-05.2006 16436592 PMC6674576

[B18] Burnashev N. Szepetowski P. (2015). NMDA receptor subunit mutations in neurodevelopmental disorders. *Curr. Opin. Pharmacol.* 20 73–82. 10.1016/j.coph.2014.11.008 25498981

[B19] Cardis R. Cabungcal J. H. Dwir D. Do K. Q. Steullet P. (2018). A lack of GluN2A-containing NMDA receptors confers a vulnerability to redox dysregulation: consequences on parvalbumin interneurons, and their perineuronal nets. *Neurobiol. Dis.* 109 64–75. 10.1016/j.nbd.2017.10.006 29024713

[B20] Cercato M. C. Vázquez C. A. Kornisiuk E. Aguirre A. I. Colettis N. Snitcofsky M. (2017). GluN1 and GluN2A NMDA receptor subunits increase in the hippocampus during memory consolidation in the rat. *Front. Behav. Neurosci.* 10:242. 10.3389/fnbeh.2016.00242 PMC5233710 28133447

[B21] Čikoš Š. Bukovská A. Koppel J. (2007). Relative quantification of mRNA: comparison of methods currently used for real-time PCR data analysis. *BMC Mol. Biol.* 8. 10.1186/1471-2199-8-113 PMC2235892 18093344

[B22] Choi J. H. Sim S. E. Kim J. Choi D. I. I. Oh J. Ye S. (2018). Interregional synaptic maps among engram cells underlie memory formation. *Science* 360 430–435. 10.1126/science.aas9204 29700265

[B23] Cohen S. J. Munchow A. H. Rios L. M. Zhang G. Ásgeirsdóttir H. N. Stackman R. W. (2013). The rodent hippocampus is essential for nonspatial object memory. *Curr. Biol.* 23 1685–1690. 10.1016/j.cub.2013.07.002 23954431 PMC3775586

[B24] Corbel C. Hernandez I. Wu B. Kosik K. S. (2015). Developmental attenuation of N-methyl-D-aspartate receptor subunit expression by microRNAs. *Neural Dev.* 10:20. 10.1186/s13064-015-0047-5 PMC4574169 26381867

[B25] Dalton G. L. Wu D. C. Wang Y. T. Floresco S. B. Phillips A. G. (2012). NMDA GluN2A and GluN2B receptors play separate roles in the induction of LTP and LTD in the amygdala and in the acquisition and extinction of conditioned fear. *Neuropharmacology* 62 797–806. 10.1016/j.neuropharm.2011.09.001 21925518

[B26] de Solis C. A. Holehonnur R. Banerjee A. Luong J. A. Lella S. K. Ho A. (2015). Viral delivery of shRNA to amygdala neurons leads to neurotoxicity and deficits in Pavlovian fear conditioning. *Neurobiol. Learn. Mem.* 124 34–47. 10.1016/j.nlm.2015.07.005 26182988 PMC4568141

[B27] Endele S. Rosenberger G. Geider K. Popp B. Tamer C. Stefanova I. (2010). Mutations in GRIN2A and GRIN2B encoding regulatory subunits of NMDA receptors cause variable neurodevelopmental phenotypes. *Nat. Genet.* 42 1021–1026. 10.1038/ng.677 20890276

[B28] Franchini L. Carrano N. Di Luca M. Gardoni F. (2020). Synaptic gluN2A-containing NMDA receptors: from physiology to pathological synaptic plasticity. *Int. J. Mol. Sci.* 21:1538. 10.3390/ijms21041538 32102377 PMC7073220

[B29] Franchini L. Stanic J. Ponzoni L. Mellone M. Carrano N. Musardo S. (2019). Linking NMDA receptor synaptic retention to synaptic plasticity and cognition. *iScience* 19 927–939. 10.1016/j.isci.2019.08.036 31518901 PMC6742927

[B30] Furini C. R. G. Nachtigall E. G. Behling J. A. K. Assis Brasil E. S. Saenger B. F. Narvaes R. F. (2020). Molecular mechanisms in hippocampus involved on object recognition memory consolidation and reconsolidation. *Neuroscience* 435 112–123. 10.1016/j.neuroscience.2020.03.047 32272151

[B31] Gambrill A. C. Barria A. (2011). NMDA receptor subunit composition controls synaptogenesis and synapse stabilization. *Proc. Natl. Acad. Sci.* 108 5855–5860. 10.1073/pnas.1012676108 21427228 PMC3078406

[B32] Gao C. Frausto S. F. Guedea A. L. Tronson N. C. Jovasevic V. Leaderbrand K. (2011). IQGAP1 regulates NR2A signaling, spine density, and cognitive processes. *J. Neurosci.* 31 8533–8542. 10.1523/jneurosci.1300-11.2011 21653857 PMC3121195

[B33] Gao C. Gill M. B. Tronson N. C. Guedea A. L. Guzmán Y. F. Al E. (2010). Hippocampal NMDA receptor subunits differentially regulate fear memory formation and neuronal signal propagation. *Hippocampus* 20 1072–1082. 10.1002/hipo.20705 19806658 PMC2891656

[B34] Gray J. A. A. Shi Y. Usui H. During M. J. J. Sakimura K. Nicoll R. A. A. (2011). Distinct modes of AMPA receptor suppression at developing synapses by GluN2A and GluN2B: single-cell NMDA receptor subunit deletion in vivo. *Neuron* 71 1085–1101. 10.1016/j.neuron.2011.08.007 21943605 PMC3183990

[B35] Hardt O. Migues P. V. Hastings M. Wong J. Nader K. (2010). PKMζ maintains 1-day- and 6-day-old long-term object location but not object identity memory in dorsal hippocampus. *Hippocampus* 20 691–695. 19806657 10.1002/hipo.20708

[B36] Holehonnur R. Phensy A. J. Kim L. J. Milivojevic M. Vuong D. Daison D. K. (2016). Increasing the GluN2A/GluN2B ratio in neurons of the mouse basal and lateral amygdala inhibits the modification of an existing fear memory trace. *J. Neurosci.* 36 9490–9504. 10.1523/jneurosci.1743-16.2016 27605622 PMC5013194

[B37] Jacobs S. Wei W. Wang D. Tsien J. Z. (2015). Importance of the GluN2B carboxy-terminal domain for enhancement of social memories. *Learn. Mem.* 22 401–410. 10.1101/lm.038521.115 26179233 PMC4509920

[B38] Kaech S. Banker G. (2007). Culturing hippocampal neurons. *Nat. Protoc.* 1 2406–2415. 10.1038/nprot.2006.356 17406484

[B39] Kandel E. R. Schwartz J. H. Jessell T. M. Siegelbaum S. A. Hudspeth A. J. Siegelbum S. A. (2012). *Principles of Neural Science.* Amsterdam: Elsevier.

[B40] Kannangara T. S. Bostrom C. A. Ratzlaff A. Thompson L. Cater R. M. Gil-Mohapel J. (2014). Deletion of the NMDA receptor GluN2A subunit significantly decreases dendritic growth in maturing dentate granule neurons. *PLoS One* 9:e103155. 10.1371/journal.pone.0103155 25083703 PMC4118862

[B41] Kim W. B. Cho J. H. (2020). Encoding of contextual fear memory in hippocampal–amygdala circuit. *Nat. Commun.* 11:1382. 10.1038/s41467-020-15121-2 PMC7069961 32170133

[B42] Kiyama Y. Manabe T. Sakimura K. Kawakami F. Mori H. Mishina M. (1998). Increased thresholds for long-term potentiation and contextual learning in mice lacking the NMDA-type glutamate receptor epsilon1 subunit. *J. Neurosci.* 18 6704–6712. 10.1523/jneurosci.18-17-06704.1998 9712642 PMC6792962

[B43] Kopp C. Longordo F. Lüthi A. (2007). Experience-dependent changes in NMDA receptor composition at mature central synapses. *Neuropharmacology* 53 1–9. 10.1016/j.neuropharm.2007.03.014 17499817

[B44] Kumar A. (2015). NMDA receptor function during senescence: implication on cognitive performance. *Front. Neurosci.* 9:473. 10.3389/fnins.2015.00473 PMC4679982 26732087

[B45] Lau C. G. Zukin R. S. (2007). NMDA receptor trafficking in synaptic plasticity and neuropsychiatric disorders. *Nat. Rev. Neurosci.* 8 413–426. 10.1038/nrn2153 17514195

[B46] Lemke J. R. Lal D. Reinthaler E. M. Steiner I. Nothnagel M. Alber M. (2013). Mutations in GRIN2A cause idiopathic focal epilepsy with rolandic spikes. *Nat. Genet.* 45 1067–1072. 23933819 10.1038/ng.2728

[B47] Lüttjohann A. Fabene P. F. van Luijtelaar G. (2009). A revised Racine’s scale for PTZ-induced seizures in rats. *Physiol. Behav.* 98 579–586. 10.1016/j.physbeh.2009.09.005 19772866

[B48] Mota Vieira M. Nguyen T. A. Wu K. Badger J. D. Collins B. M. Anggono V. (2020). An Epilepsy-Associated GRIN2A rare variant disrupts CaMKIIα phosphorylation of GluN2A and NMDA receptor trafficking. *Cell Rep.* 32:108104. 10.1016/j.celrep.2020.108104 32877683 PMC11497419

[B49] Mumby D. G. Gaskin S. Glenn M. J. Schramek T. E. Lehmann H. (2002). Hippocampal damage and exploratory preferences in rats: memory for objects, places, and contexts. *Learn. Mem.* 9 49–57. 10.1101/lm.41302 11992015 PMC155935

[B50] Paoletti P. Bellone C. Zhou Q. (2013). NMDA receptor subunit diversity: impact on receptor properties, synaptic plasticity and disease. *Nat. Rev. Neurosci.* 14 383–400. 10.1038/nrn3504 23686171

[B51] Paxinos G. Watson C. (2007). *The Rat Brain in Stereotaxic Coordinates*, 6th Edition. Amsterdam: Elsevier.

[B52] Percie, du Sert N. Ahluwalia A. Alam S. Avey M. T. Baker M. (2020). Reporting animal research: explanation and elaboration for the ARRIVE guidelines 2.0. *PLoS Biol.* 18:e3000411. 10.1371/journal.pbio.3000411 PMC7360025 32663221

[B53] Phillips R. G. LeDoux J. E. (1992). Differential contribution of amygdala and hippocampus to cued and contextual fear conditioning. *Behav. Neurosci.* 106 274–285. 10.1037/0735-7044.106.2.274 1590953

[B54] Pierson T. M. Yuan H. Marsh E. D. Fuentes-Fajardo K. Adams D. R. Markello T. (2014). GRIN2A mutation and early-onset epileptic encephalopathy: personalized therapy with memantine. *Ann. Clin. Transl. Neurol.* 1 190–198. 24839611 10.1002/acn3.39 PMC4019449

[B55] Ploquin A. Szécsi J. Mathieu C. Guillaume V. Barateau V. Ong K. C. (2013). Protection against henipavirus infection by use of recombinant adeno-associated virus-vector vaccines. *J. Infect. Dis.* 207 469–478. 10.1093/infdis/jis699 23175762 PMC7107322

[B56] Popik B. Amorim F. E. Amaral O. B. Alvares L. O. (2020). Shifting from fear to safety through deconditioning-update. *Elife* 9:e51207. 10.7554/eLife.51207 PMC7021486 31999254

[B57] Punnakkal P. Dominic D. (2018). NMDA receptor GluN2 subtypes control epileptiform events in the hippocampus. *Neuro Mol. Med.* 20 90–96. 10.1007/s12017-018-8477-y 29335819

[B58] Rodriguez A. Zhang H. Klaminder J. Brodin T. Andersson P. L. Andersson M. (2018). ToxTrac: a fast and robust software for tracking organisms. *Methods Ecol. Evol.* 9 460–464. 10.1111/2041-210x.12874

[B59] Sacchetti B. Lorenzini C. A. Baldi E. Tassoni G. Bucherelli C. (1999). Auditory thalamus, dorsal hippocampus, basolateral amygdala, and perirhinal cortex role in the consolidation of conditioned freezing to context and to acoustic conditioned stimulus in the rat. *J. Neurosci.* 19 9570–9578. 10.1523/jneurosci.19-21-09570.1999 10531459 PMC6782906

[B60] Samokhina E. Samokhin A. (2018). Neuropathological profile of the pentylenetetrazol (PTZ) kindling model. *Int. J. Neurosci.* 128 1086–1096. 10.1080/00207454.2018.1481064 29792126

[B61] Sans N. Petralia R. S. Wang Y. X. Blahos J. Hell J. W. Wenthold R. J. (2000). A developmental change in NMDA receptor-associated proteins at hippocampal synapses. *J. Neurosci.* 20 1260–1271. 10.1523/jneurosci.20-03-01260.2000 10648730 PMC6774158

[B62] Sanz-Clemente A. Nicoll R. A. Roche K. W. (2013). Diversity in NMDA receptor composition: many regulators, many consequences. *Neuroscientist* 19 62–75. 10.1177/1073858411435129 22343826 PMC3567917

[B63] Sefil F. Kahraman I. Dokuyucu R. Gokce H. Ozturk A. Tutuk O. (2014). Ameliorating effect of quercetin on acute pentylenetetrazole induced seizures in rats. *Int. J. Clin. Exp. Med.* 7 2471–2477. 25356099 PMC4211749

[B64] Sepulveda F. J. Bustos F. J. Inostroza E. Zúñiga F. A. Neve R. L. Montecino M. (2010). Differential roles of NMDA receptor subtypes NR2A and NR2B in dendritic branch development and requirement of RasGRF1. *J. Neurophysiol.* 103 1758–1770. 10.1152/jn.00823.2009 20107120

[B65] Shipton O. A. Paulsen O. Paulsen O. (2014). GluN2A and GluN2B subunit-containing NMDA receptors in hippocampal plasticity. *Philos. Trans. R. Soc. Lond. B. Biol. Sci.* 369:20130163. 10.1098/rstb.2013.0163 24298164 PMC3843894

[B66] Sibarov D. A. Bruneau N. Antonov S. M. Szepetowski P. Burnashev N. Giniatullin R. (2017). Functional properties of human NMDA receptors associated with epilepsy-related mutations of GluN2A subunit. *Front. Cell. Neurosci.* 11:155. 10.3389/fncel.2017.00155 PMC5447064 28611597

[B67] Strehlow V. Heyne H. O. Vlaskamp D. R. M. Marwick K. F. M. Rudolf G. de Bellescize J. (2019). GRIN2A -related disorders: genotype and functional consequence predict phenotype. *Brain* 142 80–92. 30544257 10.1093/brain/awy304 PMC6308310

[B68] Swanger S. A. A. Chen W. Wells G. Burger P. B. B. Tankovic A. Bhattacharya S. (2016). Mechanistic insight into NMDA receptor dysregulation by rare variants in the GluN2A and GluN2B agonist binding domains. *Am. J. Hum. Genet.* 99 1261–1280. 10.1016/j.ajhg.2016.10.002 27839871 PMC5142120

[B69] Vedeler A. Hollas H. Grindheim A. K. Raddum M. A. (2012). Multiple roles of annexin A2 in post-transcriptional regulation of gene expressio. *Curr. Protein Pept. Sci.* 13 401–412. 10.2174/138920312801619402 22708494

[B70] Warburton E. C. Barker G. R. I. Brown M. W. (2013). Investigations into the involvement of NMDA mechanisms in recognition memory. *Neuropharmacology* 74 41–47. 10.1016/j.neuropharm.2013.04.013 23665343 PMC3895175

[B71] Wenthold R. J. Prybylowski K. Standley S. Sans N. Petralia R. S. (2003). T Rafficking Of Nmda R Eceptors. *Annu. Rev. Pharmacol. Toxicol.* 43 335–358. 12540744 10.1146/annurev.pharmtox.43.100901.135803

[B72] Wigström H. Gustafsson B. (1986). Postsynaptic control of hippocampal long-term potentiation. *J. Physiol.* 81 228–236. 2883309

[B73] Wu X. H. Ding M. P. Zhu-Ge Z. B. Zhu Y. Y. Jin C. Chen Z. (2006). Carnosine, a precursor of histidine, ameliorates pentylenetetrazole-induced kindled seizures in rat. *Neurosci. Lett.* 400 146–149. 10.1016/j.neulet.2006.02.031 16515835

[B74] Yashiro K. Philpot B. D. (2008). Regulation of NMDA receptor subunit expression and its implications for LTD, LTP, and metaplasticity. *Neuropharmacology* 55 1081–1094. 10.1016/j.neuropharm.2008.07.046 18755202 PMC2590778

[B75] Yeh S. H. Hung J. J. Gean P. W. Chang W. (2008). Hypoxia-inducible factor-1α protects cultured cortical neurons from lipopolysaccharide-induced cell death via regulation of NR1 expression. *J. Neurosci.* 28 14259–14270. 10.1523/jneurosci.4258-08.2008 19109507 PMC6671468

[B76] Yuan H. Hansen K. B. Zhang J. Pierson T. M. Markello T. C. Fuentes Fajardo K. V. (2014). Functional analysis of a de novo GRIN2A missense mutation associated with early-onset epileptic encephalopathy. *Nat. Commun.* 5:3251. 10.1038/ncomms4251 PMC3934797 24504326

[B77] Zeng F. Ma X. Zhu L. Xu Q. Zeng Y. Gao Y. (2019). The deubiquitinase USP6 affects memory and synaptic plasticity through modulating NMDA receptor stability. *PLoS Biol.* 17:e3000525. 10.1371/journal.pbio.3000525 31841517 PMC6913916

[B78] Zhang X. M. Yan X. Y. Zhang B. Yang Q. Ye M. Cao W. (2015). Activity-induced synaptic delivery of the GluN2A-containing NMDA receptor is dependent on endoplasmic reticulum chaperone Bip and involved in fear memory. *Cell Res.* 25 818–836. 10.1038/cr.2015.75 26088419 PMC4493282

[B79] Zukin R. S. Bennett M. V. L. (1995). Alternatively spliced isoforms of the NMDARI receptor subunit. *Trends Neurosci.* 18 306–313. 10.1016/0166-2236(95)93920-s 7571011

