# Supplementary material for: Reduced Expression of Hippocampal GluN2A-NMDAR Increases Seizure Susceptibility and Causes Deficits in Contextual Memory
Source: Front Neurosci. 2021 Apr 9;15:644100. doi: 10.3389/fnins.2021.644100 (PMC8064689; doi:10.3389/fnins.2021.644100)
Supplement: Supplementary file 1 [file Table_1.DOCX]

Supplementary Material

# Supplementary Figure

**
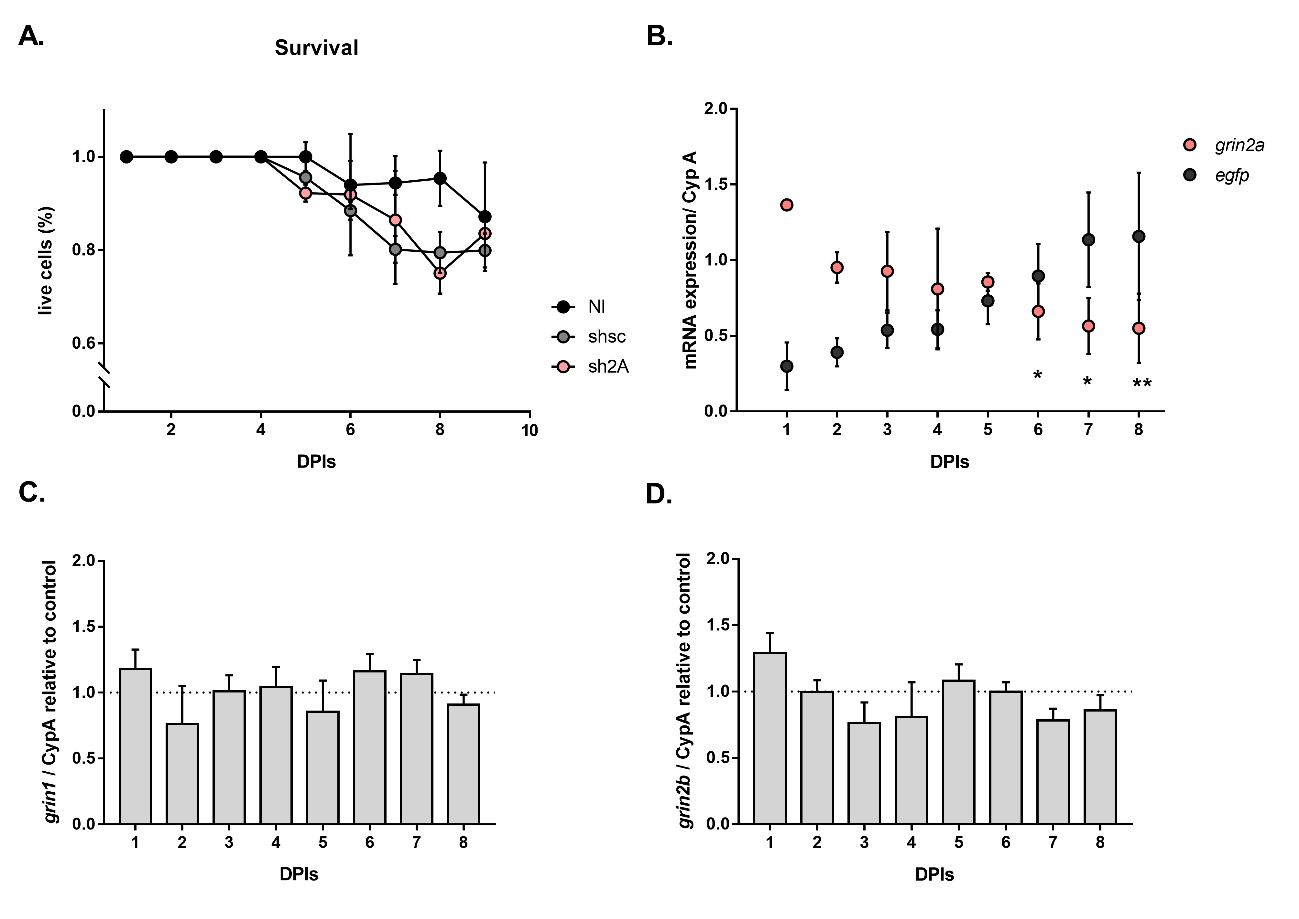
**

**Supplementary Figure 1.** **Analysis of the AAV-transduced primary neuronal cultures at different days post-infection (DPIs).** **A.** The shsc- and shGluN2A-transduced cultures presented similar cell viability along all the DPIs analyzed by Hoestch staining. At 8 DPIs, there was a little difference between shsc- and shGluN2A-transduced cultures compared to NI cultures. No significant differences were detected between shsc-, sh2A-transduced and NI cultures on all the analyzed days (*P<0.05, two-way ANOVA, Sidak post-test). **B.** mRNA relative expression of *egfp* (black) and *grin2a* (pink) from 1 to 8 DPIs in AAV-shGluN2A-transduced neuronal cultures. *Egfp* expression increased progressively at the same time as *grin2a* mRNA levels decreased. *grin2a* levels became significantly decreased at 6 DPI (* P < 0.05, one-way ANOVA, Tukey post-test, n=5) and continues decreasing up to 8 DPI (* P < 0.05, ** P < 0.01, one-way ANOVA, Tukey post-test, n=3). **C.** GluN1 (*grin1*) and **D.** GluN2B mRNA (*grin2b*) remained similar to the shsc-transduced cultures along the days (one-way ANOVA, Tukey post-test). Line represents shsc relative control levels. mRNA levels measured by RT-qPCR, using *CypA* as housekeeping. Data are represented as mean ± SEM. N=3 independent cultures for all the DPI, N=5 independent cultures for 6 DPI, and N=4 for 7 DPI.
